# Acceptance, initial trust formation, and human biases in artificial intelligence: Focus on clinicians

**DOI:** 10.3389/fdgth.2022.966174

**Published:** 2022-08-23

**Authors:** Avishek Choudhury, Safa Elkefi

**Affiliations:** ^1^ Industrial and Management Systems Engineering, Benjamin M. Statler College of Engineering and Mineral Resources, West Virginia University, Morgantown, WV, United States; ^2^ School of Systems and Enterprises, Stevens Institute of Technology, Hoboken, NJ, United States

**Keywords:** trust & distrust, artificial intelligence, healthcare, patient safety, technology acceptance, communication, human biases

## Introduction

Healthcare systems have benefited from technology use and development. Different technologies have augmented the healthcare system at different capacities and consequently have advocated for patient safety. *Artificial Intelligence (AI) is one such emerging technology that can extract latent information from a plethora of data to formulate actionable knowledge based on pre-defined or adaptive algorithms.* Unlike other healthcare technologies, AI has not been wholeheartedly welcomed by the healthcare community and has often gained an overall negative perception. We acknowledge that healthcare AI, like any other technology, comes with certain drawbacks, but its potential to augment healthcare systems and care quality is a significant factor encouraging its integration into the clinical workflow.

Research around healthcare AI can be categorized into two groups, one that promises multiple benefits of the AI (1, 2) and the other that criticizes the technology (3). Although both the body of work is crucial and noteworthy, not much evidence exists that can confirm AIs' negative impact on patient safety or health outcome. Studies have demonstrated AI's potential in multiple healthcare domains, including the dentistry (4), pediatric (5), and geriatric and adult populations (6). Given the evidence and potential of AI, it is now the time to emphasize improving AI acceptance and trust among clinicians and healthcare organizations. Currently, several studies, not limited to healthcare, are directed to resolve the problem of distrust in AI, where the trend is to achieve a calibrated trust in the technology (7, 8). Trust in AI can be defined as the tendency of the user to rely on AI recommendation and willingly be vulnerable to the technology. Thus far, most of the studies addressing AI trust have assumed trust as a construct that varies overtime and have explored the role of fairness (9), interpretability, privacy (10, 11), and algorithmic awareness (12) in shaping users' trust in AI. Other factors such as their interaction with the technology, AI accuracy, data quality, and biases, have been considered to significantly impact user trust in AI. However, it is important to understand that these factors are only experienced by the user once they use the technology. For example, to realize the accuracy, transparency, or usability of a given AI system, the user has to first use the technology. What is missing in the literature is to capture the factors that would initially motivate the user to use the AI system for the very first time. That is, to explore the potential cognitive human factors responsible for shaping clinicians' initial trust in AI. Since clinicians typically resist and hold a negative perception of the technology, it is essential to understand how initial trust in AI is formed.

We define initial trust as a binary (trust = 1 or distrust = 0) latent construct that can be confirmed if the user, without any obligation or influence, acts upon/accepts AI recommendations without having any prior experience with the technology. In other words, initial trust in AI is a measure of the extent to which a clinician is willing to take clinical action or decision based on the information generated by the AI technology, provided free will of the clinician and no prior experience with the technology. Initial trust in AI is a significant precursor to its use and gradual acceptance as a useful healthcare technology. In this article, we portray initial trust in AI as a function of multiple factors such as information relevance, patient risk, and the mental model or perception of the clinician. We also discuss how AI can improve healthcare communication.

## Initial trust formation in healthcare artificial intelligence: Clinicians' perspective

It is important to understand that an AI system that is implemented into a clinical workflow is extensively tested and approved by the concerned authorities ensuring its accuracy and overall performance. What's missing is understanding why clinicians hesitate to adopt AI and the factors determining their initial trust formation in the technology. In general, trust in AI fluctuates with prior experience and repeated interactions (iterative) with the technology, but the initial trust (i.e., trust in an unfamiliar entity without prior experience) is formed through assumptions, perceptions, and quick inferences about the trustee from a limited information (13). Understanding the initial trust formation is essential to initiate AI use and acceptance in the healthcare domain.

**Figure 1:**
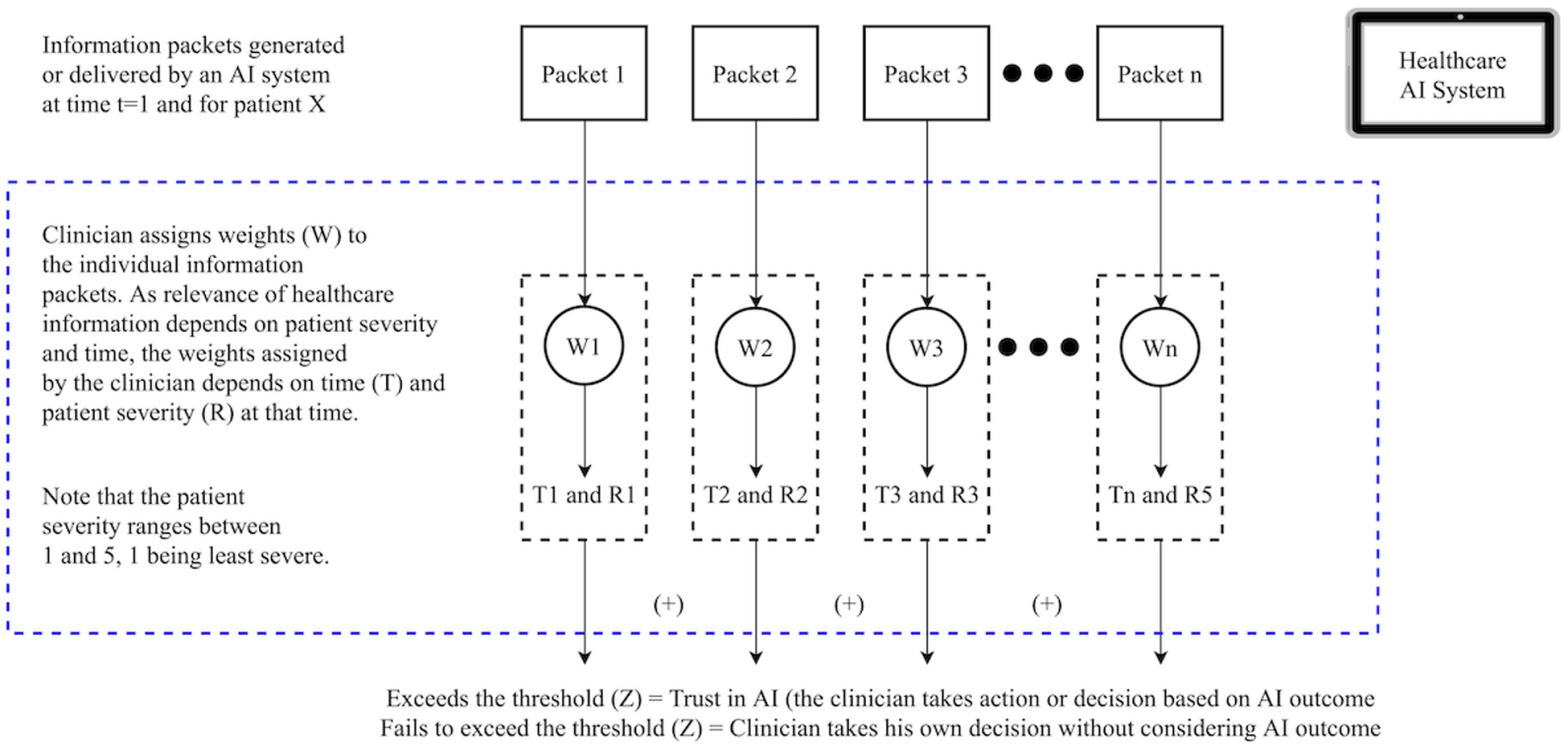
Clinicians’ initial trust formation in artificial intelligence.

Literature has identified several important factors that shape users’ trust in AI, including AI biases, data quality, reliability, and explainability (14). But these factors primarily depend on the user perceptions and situations, cannot be measured with a numeric standard value. Thus, it is important to incorporate human factors consideration. Whether any clinician (first-time AI user) will place their initial trust in the technology depends on whether the clinician thinks that the information (recommendations) generated by the AI will have clinical relevance and will be applicable to a given context. Figure 1 illustrates a hypothetical and a general scenario where an AI system embedded in the electronic health record produces a set of recommendations for a clinician.

The recommendation generated by the AI comprises multiple packets of information $\left(I=\sum_{1}^{n}\left(\mathrm{Packet}\right)\right)$. After receiving this onscreen information (I), the clinician assigns weight (W) to each piece of information. The weights (W) assigned to each information depend on time (T) and patient severity (R) $\left(W_{n}=f\left(Tn,Rn\right)\right)$. Certain information can be crucial at a given point in time and for a certain patient type. Note that patient severity (R) is a measure of their health status (deviation from the healthy baseline. For patient acuity, R can range from 1 to 5).

To ensure clinicians' initial trust in AI (i.e., Initial Trust = 1), the summation of the product of weights and information packet (*W_n_I_n_*) should exceed a given threshold (Z), i.e., (*W_n_I_n_* > Z where $W_{n}=\sum_{1}^{n}\left(W\right)$ and $I=\sum_{1}^{n}\left(\mathrm{Packet}\right)$). The threshold (Z) depends on several human factors, including the clinician's experience (Ex), expertise (Ep), situation awareness (SA), and cognitive workload (WL). In other words, Z=f(Ex,Ep,SA,WL). Fundamentally, users' (clinicians') initial trust depends on the information generated by a given AI technology and not the technology or its functioning per se.

## Human biases prevent acceptance of artificial intelligence

AI biases have been extensively discussed in the literature, where studies have acknowledged how skewed data can induce bias in AI. However, the inherent and latent biases in human users are not well recognized in the context of healthcare AI. Human (clinicians') biases are a strong determinant of initial trust in AI and can hinder its acceptance in healthcare.

In the context of specialist doctors evaluating AI-generated diagnoses or insights, we may observe a complex interplay of expert bias and the limitations inherent in highly specialized knowledge. These doctors, while exceedingly knowledgeable and skilled in their specific domains, may exhibit a form of overconfidence bias when confronting information that falls outside their immediate area of expertise. This is not due to a lack of competence, but rather a natural consequence of deep specialization: as one's expertise becomes more focused, awareness of developments and data patterns in broader or tangentially related fields may diminish.

This situation is further compounded by confirmation bias, where specialists might prefer information or interpretations that align with their existing knowledge and experience, leading to potential skepticism or undervaluation of AI insights that present novel correlations or findings outside their specialization.

Recognizing and addressing such bias is crucial for the effective integration of AI in healthcare, ensuring that the complementary strengths of human expertise and advanced algorithms are optimally utilized.

## Healthcare communication and artificial intelligence

Communication is an essential process in the healthcare industry. Communication between clinicians and AI can foster patient safety and quality of care. However, under excessive workload and chaos, clinicians may miss some of the critical information projected by the AI system or fail to comprehend its actual meaning. To benefit from AI systems and develop the initial trust, ensuring a perception of shared understanding between AI and clinician, is essential. Once initial trust is established, AI integration into the clinical workflow can play an essential role in improving the clinical communication (15). For example, a speech recognition device powered by AI can detect distinct vocal changes associated with specific health conditions (16). Vocal analysis and speech recognition can help doctors make informed decision-making and encourage personalized care.

The typical notion about AI is that the technology will replace clinicians and reduce interpersonal touch. However, in reality, AI can increase doctor-patient interaction time. Currently, clinician-patient interaction is hindered by excessive clinical workload. Clinicians, during patient visits, spend a significant amount of time taking notes and simultaneously updating the electronic health records system. In this scenario, patients typically feel they are not being listened to. AI technologies (e.g., Voice AI) can eliminate excessive clinical workload by automating taking clinical notes (17). AI can also enter each item into the appropriate field or section of the EHR (17). AI can also play an essential role in coordinating between doctors and patients, and improve information exchange between the stakeholders by facilitating their work and controlling the risks related to clinical tasks (18). AI can also be used to simulate possible scenarios that can help teach nurses how to hone their communication skills before going on to clinical placements using unlimited training attempts in a safe and secure environment (19).

## Discussion and conclusion

In this article, we reemphasize the importance of initial trust formation in AI, clinicians' perception, and how they assign relevance to AI outputs for a given patient. Additionally, we also discuss the role and importance of considering cognitive biases to encourage AI acceptance and how AI can augment healthcare communication. Much of the research around healthcare AI has been designed to improve AI transparency, an important component. However, algorithmic transparency will not suffice or ensure AI acceptance. Not only does making the fundamental function of AI systems transparent may cause proprietary issues, but it can also consume clinicians' working memory because understanding AI algorithms are difficult. Having the complex AI functioning along with clinical procedural information stored in long-term memory while attending to critical patients can be overwhelming and not feasible for many clinicians.

When automated diagnostic systems are used in real-life clinics, it is most likely as assistant or recommendation systems, where the AI system provides information to clinicians or patients as a second opinion. However, if the suggestions made by AI are entirely data-driven without accounting for user's opinion, users could get skewed toward or against the suggestion of the AI system leading to automation surprises or biases. Therefore, the next push for researchers should be to move AI research out from solely model development into socio-technical systems research and effectively use human factors and psychological principles emphasizing its ecological validity. It is important to understand that a clinical environment is not inherently equivalent to predictable mechanical systems and needs a systematic approach where users' perception is critical. In fact, more realistically, the acceptance of AI into the inpatient clinical workflow entirely depends on how doctors and nurses perceive this technology – their intent to use AI and their initial trust in the technology.
