# Corrigendum: Acceptance, initial trust formation, and human biases in artificial intelligence: focus on clinicians

**DOI:** 10.3389/fdgth.2024.1334266

**Published:** 2024-02-28

**Authors:** Avishek Choudhury, Safa Elkefi

**Affiliations:** ^1^ Industrial and Management Systems Engineering, Benjamin M. Statler College of Engineering and Mineral Resources, West Virginia University, Morgantown, WV, United States; ^2^ School of Systems and Enterprises, Stevens Institute of Technology, Hoboken, NJ, United States

**Keywords:** trust & distrust, artificial intelligence, healthcare, patient safety, technology acceptance, communication, human biases

A Corrigendum on Acceptance, initial trust formation, and human biases in artificial intelligence: focus on clinicians By Choudhury A, Elkefi S (2022). Front. Digit. Health 4:966174. doi: 10.3389/fdgth.2022.966174

**Incorrect Reference**

In the published article, the reference for the Dunning-Kruger effect is no longer valid as the authors have removed it from the text. The original reference was:

15. Dunning D. The Dunning–Kruger effect: On being ignorant of one’s own ignorance. In: J. M. Olson & M. P. Zanna, editors. Advances in experimental social psychology. San Diego, CA: Elsevier (2011). p. 247–296.

**Text Correction**

In the published article, there was an error in the section **Human biases prevent acceptance of artificial intelligence**, paragraph 2. The phenomena discussed below is not the Dunning-Kruger effect but expert bias. The paragraph previously stated:

“Clinicians often resist AI integration into their workflow. Their skepticism toward AI builds upon a few presumptions, where lack of initial trust in technology plays a significant role. The Dunning-Kruger effect explains why clinicians refrain from trusting AI systems. According to the Dunning-Kruger effect, people with expertise in a specific field (specialist doctor) often overestimate their own competence in that domain and perceive their own opinion or judgment over anything else (15), including AI. It is common for clinical experts (senior physicians) to express confirmation bias and ignore AI's recommendation whenever it contradicts their presumption/judgment. Thus far, several initiatives have been taken by national and global authorities to regulate, standardize, and improve AI. However, critical factors such as cognitive biases and user perceptions require further exploration.”

The corrected paragraphs appear below:

“In the context of specialist doctors evaluating AI-generated diagnoses or insights, we may observe a complex interplay of expert bias and the limitations inherent in highly specialized knowledge. These doctors, while exceedingly knowledgeable and skilled in their specific domains, may exhibit a form of overconfidence bias when confronting information that falls outside their immediate area of expertise. This is not due to a lack of competence, but rather a natural consequence of deep specialization: as one's expertise becomes more focused, awareness of developments and data patterns in broader or tangentially related fields may diminish.

This situation is further compounded by confirmation bias, where specialists might prefer information or interpretations that align with their existing knowledge and experience, leading to potential skepticism or undervaluation of AI insights that present novel correlations or findings outside their specialization.

Recognizing and addressing such bias is crucial for the effective integration of AI in healthcare, ensuring that the complementary strengths of human expertise and advanced algorithms are optimally utilized.”

The authors apologize for this error and state that this does not change the scientific conclusions of the article in any way. The original article has been updated.

